# Lattice Boltzmann-based numerical analysis of nanofluid natural convection in an inclined cavity subject to multiphysics fields

**DOI:** 10.1038/s41598-022-09320-8

**Published:** 2022-04-01

**Authors:** Muhammad Ibrahim, Abdallah S. Berrouk, Tareq Saeed, Ebrahem A. Algehyne, Vakkar Ali

**Affiliations:** 1 Mechanical Engineering Department, Khalifa University of Science and Technology, SAN Campus, PO Box 127788, Abu Dhabi, United Arab Emirates; 2 Center for Catalysis and Separation, Khalifa University of Science and Echnology, PO Box 127788, Abu Dhabi, United Arab Emirates; 3 Nonlinear Analysis and Applied Mathematics (NAAM)-Research Group, Department of Mathematics, Faculty of Science, King Abdulaziz University, P.O. Box 80203, Jeddah, 21589 Saudi Arabia; 4 Department of Mathematics, Faculty of Science, University of Tabuk, P.O.Box741, Tabuk, 71491 Saudi Arabia; 5 Nanotechnology Research Unit (NRU), Faculty of Science, University of Tabuk, Tabuk, Saudi Arabia; 6 Department of Mechanical and Industrial Engineering, College of Engineering, Majmaah University, Al-Majmaah, 11952 Saudi Arabia

**Keywords:** Mechanical engineering, Computational science

## Abstract

This research conducts a study of natural convection heat transfer (NCHT) in a nanofluid under a magnetic field (MF). The nanofluid is in a cavity inclined at an angle of 45°. The MF can take different angles between 0° and 90°. Radiative heat transfer is present in the cavity in volumetric form. There are two hot semicircles, similar to two half-pipes, on the bottom wall. The top wall is kept cold. The side walls and parts of the bottom wall, except the pipes, have been insulated. The lattice Boltzmann method has been used for the simulation. The studied parameters are the Rayleigh number (in the range 10^3^–10^6^), magnetic field angle, radiation parameter (in the range 0–2), and nanoparticle volume fraction (in the range 0–5%). The generated entropy has been studied as the NCHT. The results indicate that adding nanoparticles improves heat transfer rate (HTR). Moreover, the addition of volumetric radiation to the cavity enhances the Nusselt number by 54% and the generated entropy by 12.5%. With an augmentation in the MF angle from 0° to 90°, HTR decreases and this decrease is observed mostly at higher Rayleigh numbers. An augmentation in the Ra increases NCHT and entropy generation. Indeed, a rise in the Ra from 10^3^ to 10^6^ increases HTR by almost sixfold.

## Introduction

Improving HTR can reduce energy consumption in buildings^1–4^, energy-intensive industries^5–9^, and desalinators^10–14^ and generally improve performance^15,16^. NCHT with radiation occurs in some industrial and laboratory equipment^17,18^. Many research works conducted on NCHT, especially in closed cavities, have ignored RHT^19^. On the other hand, the presence of radiation is significant in some closed cavity applications. An instant of this case is solar collectors^20–22^. Furthermore, the operating temperature is very high in some closed cavity industrial applications. Furnaces and boilers are examples of high-temperature applications of closed cavities. Consequently, some researchers have considered NCHT with radiation inside closed cavities^23–30^. In one of these studies, Karimipour et al.^31^ analyzed NCHT with radiation. They used a rectangular cavity and examined the effect of the cavity angle. They compared the convective and radiative heat transfer levels and plotted average Nu graphs for dissimilar nanoparticle volume fractions. In another paper, Sheikholeslami et al.^32^ studied NCHT with volumetric radiation. They used water-iron oxide nanofluid and pure water for their work. They also examined NCHT with radiation and investigated HTR for different volumetric radiation levels.

Using nanofluids instead of pure fluids can contribute to improving HTR^33–37^. Nanoparticles have been used in numerous studies^23,38–43^. This is because nanofluids have a greater heat conductivity coefficient than pure fluids. In one of these studies, Rahimi et al.^44^ studied NCHT in a L-shaped cavity. They used a hybrid nanofluid for their work. Their results show intensification in NCHT with the addition of nanoparticles and a rise in the Ra. Another modification made by researchers on closed cavities to investigate its effect is changing the angle of the cavity. Sheremet et al.^45^ considered the effect of the cavity angle on the NCHT of water-alumina nanofluid inside a square cavity. The cavity angle can have different impacts on the dependency of HTR on the shape and conditions of the cavity. Under different conditions, the cavity has stronger convection at different angles.

The presence of a MF is inevitable in various real-life applications^46–49^; hence, it is needed to study the influence of the MF on the cavity. In some of these papers, the MF has different angles, and the impact of the MF angle has been investigated. Akter et al.^50^ considered the NCHT of air inside an asymmetric cavity. They subjected the cavity to a constant MF. The MF is at an angle with respect to the horizontal. Their results indicate the unsuitability of the MF for vortex formation inside the cavity.

The study of entropy generation simultaneously with HTR has drawn the attention of numerous researchers in recent years. One can investigate the dissipation during a process according to the second law of thermodynamics by studying the entropy generation during that process. Pordanjani et al.^24^ simultaneously studied entropy generation and NCHT inside a rectangular cavity. They examined the entropy generated by varying different parameters. In the end, they discovered that the factors that intensify HTR can also increase entropy generation in the cavity. In another paper, Alsabery et al.^51^ studied generated entropy by water-alumina nanofluid in a square cavity with an obstacle in its middle. They used the finite difference method for their simulation. Their results show a rise in entropy generation with an improvement in NCHT in the cavity.

An application of NCHT is in solar collectors^20^. NCHT and RHT are present simultaneously in these collectors. In some of these applications, the existence of a MF is inevitable, and it is necessary to study its impact on HTR. To better study the performance of the cavity, one needs to investigate also the generated entropy in the system. Accordingly, the RHT and NCHT of a nanofluid in a square chamber have been simulated in this paper using the LBM. The cavity is subjected to a MF at different angles. The chamber is inclined, and the entropy generation has also been studied. There are two semicircles in the form of two hot half-pipes that heat the cavity. The studied parameters are the Ra, the radiation parameter, the nanoparticle volume fraction, and the MF angle. The innovation of the present work is the special problem geometry and the simultaneous study of HTR and entropy in the presence of radiation and an oblique MF. The novelty of the article is the specific geometry of the chamber along with the method of solving it.

## Problem statement

**Figure 1:**
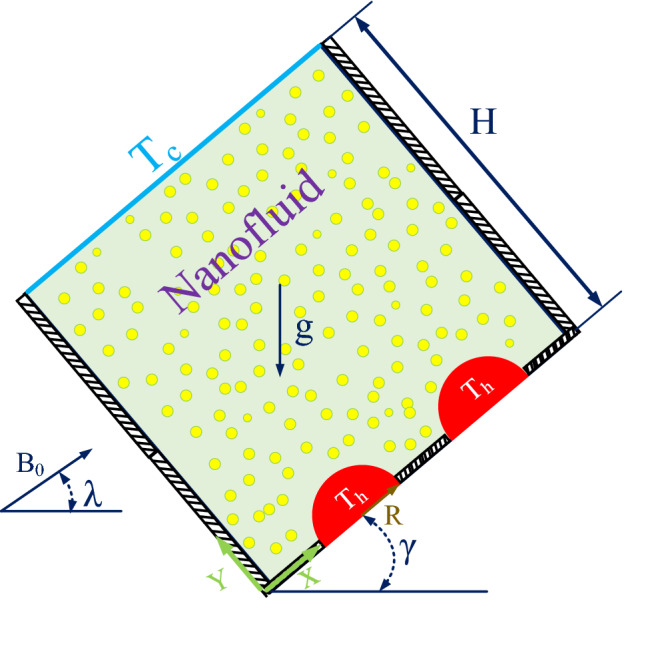
The geometry of the problem.

The problem schematic is a square with side H, as shown in Fig. 1. This two-dimensional square, which is called a closed cavity, has a 45° angle with the horizontal. The cavity is containing the Al_2_O_3_–Water nanofluid. A MF with an intensity of B_0_ and an angle of $\lambda$ with the horizon is incident on the chamber, and two hot, pipe-shaped semicircles at a temperature of T_h_ are at the bottom wall. Moreover, the top wall is cold and at a temperature of T_c_. The other parts of the bottom wall and the side walls have been insulated.

### Lattice Boltzmann method

The LBM instigated from the lattice gas automata method (LGAM). The LGAM presents a model of the collision of virtual particles onto an ordered lattice^52–57^. The initial LBM was created by McNamara and Zanetti^57^ to address one of the main issues of the LGAM, namely the statistical noise issue. A while later, the LBM was found to be able to naturally resolve most of the other issues of the LGAM. Therefore, the LBM rapidly became an independent research topic, and the remaining issues of the LGAM were resolved one by one. Using the Boltzmann condition, the following modifications in the probability of dissemination work may be communicated:

1 $$f_{\alpha } \left( {\vec{x} + \vec{e}_{\alpha } \delta t,t + \delta t} \right) - f_{\alpha } \left( {\vec{x},t} \right) = - \frac{1}{{\tau_{v} }}\left( {f_{\alpha } \left( {\vec{x},t} \right) - f_{\alpha }^{eq} \left( {\vec{x},t} \right)} \right)$$

where addresses the balance dissemination work, $\tau_{v}$ is the relaxation time, and $\vec{e}_{\alpha }$ is the velocity vector of the particles in the direction of $\alpha$.

where $f_{\alpha } \left( {\vec{x},t} \right)$ denotes the balance dispersion work, $\tau_{v}$ denotes the relaxation time, and $\vec{e}_{\alpha }$ denotes the particle velocity vector in the direction of $\alpha$.

2 $$f_{\alpha }^{eq} \left( {\vec{x},t} \right) = \omega_{\alpha } \rho \left[ {1 + \frac{3}{{c^{2} }}\left( {\vec{e}_{\alpha } \cdot \vec{u}} \right) + \frac{9}{{2c^{2} }}\left( {\vec{e}_{\alpha } \cdot \vec{u}} \right)^{2} - \frac{3}{{2c^{2} }}\vec{u}^{2} } \right]$$

3 $$\vec{e}_{\alpha } = \left\{ {\begin{array}{*{20}l} {\left( {0,0} \right),} \hfill & {\quad \alpha = 9} \hfill \\ {\left( {\cos \left[ {\frac{{\left( {\alpha - 1} \right)\pi }}{2}} \right],\sin \left[ {\frac{{\left( {\alpha - 1} \right)\pi }}{2}} \right]} \right)c,} \hfill & {\quad \alpha = 1,2,3,4} \hfill \\ {\sqrt 2 \left( {\cos \left[ {\frac{{\left( {\alpha - 5} \right)\pi }}{2} + \frac{\pi }{4}} \right],\sin \left[ {\frac{{\left( {\alpha - 5} \right)\pi }}{2} + \frac{\pi }{4}} \right]} \right)c,} \hfill & {\quad \alpha = 5,6,7,8} \hfill \\ \end{array} } \right.$$

In the above equation, $c = \frac{\delta x}{{\delta t}}$, where $\delta x$ and $\delta t$ respectively represent the step size and time step of the lattice, respectively. Moreover, $\omega_{\alpha }$ is the weight function, which, in the $D_{2} Q_{9}$ model, equals the following:

4 $$\vec{e}_{\alpha } = \left\{ {\begin{array}{*{20}l} {4/9,} \hfill & {\quad \alpha = 9} \hfill \\ {1/9,} \hfill & {\quad \alpha = 1,2,3,4} \hfill \\ {1/36,} \hfill & {\quad \alpha = 5,6,7,8} \hfill \\ \end{array} } \right.$$

In the study of NCHT in a nanofluid under a MF, the buoyancy force and the uniform MF are the forces exerted on the nanofluid. To apply them, the following term will be added:

5 $$F_{\alpha } = \omega_{\alpha } F\frac{{\vec{e}_{\alpha } }}{{c_{s}^{2} }}$$

6 $$F = F_{x} + F_{y}$$

7 $$F_{x} = 3\omega_{i} \rho \left[ {g\beta \left( {T - T_{m} } \right)sin\gamma } \right]$$

8 $$F_{y} = 3\omega_{i} \rho \left[ {g\beta \left( {T - T_{m} } \right)cos\gamma - \frac{{{\sigma B}_{0}^{2} }}{{\uprho }}{\text{v}}} \right]$$

$\beta$, $g$, ${\text{B}}_{0}$, and $\gamma$ respectively denote the volumetric thermal expansion coefficient, gravitational acceleration, MF, and cavity angle. Finally, the following equations may be used to compute the density and macroscopic velocity:

9 $$\rho = \mathop \sum \limits_{\alpha = 1}^{9} f_{\alpha }$$

10 $$\rho {\vec{\text{u}}} = \mathop \sum \limits_{\alpha = 1}^{9} \vec{e}_{\alpha } f_{\alpha }$$

The lattice viscosity will be calculated as follows:

11 $$\nu = \left( {\tau_{v} - 0.5} \right)c^{2} /3$$

The velocity relaxation time must always be larger than 0.5 since negative viscosity has no physical value. All simulations in this study used a velocity relaxation time of 0.56. The changes in the temperature distribution function are defined using the Boltzmann equation as follows^52^:

12 $${\text{g}}_{{\upalpha }} \left( {\vec{x} + \vec{e}_{\alpha } \delta t,t + \delta t} \right) - {\text{g}}_{\alpha } \left( {\vec{x},t} \right) = - \frac{1}{{\tau_{c} }}\left( {{\text{g}}_{\alpha } \left( {\vec{x},t} \right) - {\text{g}}_{\alpha }^{eq} \left( {\vec{x},t} \right)} \right)$$

In the above equation, $\tau_{c}$ is the non-dimensional energy relaxation time. In addition, ${\text{g}}_{\alpha }^{eq}$ is the temperature equilibrium distribution function and is known as follows:

13 $${\text{g}}_{\alpha }^{eq} \left( {\vec{x},t} \right) = \omega_{{\upalpha }} T\left( {1 + \frac{3}{{c^{2} }}\left( {\vec{e}_{\alpha } ,{\vec{\text{u}}}} \right)} \right)$$

The following is how the heat from radiation is inserted into the energy equation:

14 $$g_{\alpha } = \omega_{\alpha } G\frac{{\vec{e}_{\alpha } }}{{c_{s}^{2} }}$$

15 $$G = \omega_{{\upalpha }} T\left( {\frac{1}{{\rho C_{P} }}\frac{{\partial q_{r} }}{\partial y}} \right)$$

16 $$q_{r} = - \frac{4}{3}\frac{{\sigma_{e} }}{{\beta_{R} }}\frac{{\partial T^{4} }}{\partial y},\;T^{4} = 4T_{c}^{3} T - 3T_{c}^{4}$$

The macroscopic temperature value is finally determined as follows:

17 $$T = \mathop \sum \limits_{\alpha = 1}^{9} {\text{g}}_{{\upalpha }}$$

As a result, the lattice diffusion coefficient is calculated as this:

18 $$\alpha = \left( {\tau_{C} - 0.5} \right)c^{2} /3$$

Because a negative diffusion coefficient is physiologically nonsensical, the energy relaxation time must always be larger than 0.5, just as the velocity relaxation time. In this study, the value of this parameter is assumed to be 0.58.

The equations of the LBM are run in two stages: collision and streaming. Collision step: The collision step begins infinitely soon before the collision and ends indefinitely soon after. The following is an example of how it may be expressed:

19 $$\tilde{f}_{\alpha } \left( {\vec{x},t} \right) = f_{\alpha } \left( {\vec{x},t} \right) - \frac{1}{{\tau_{v} }}\left( {f_{\alpha } \left( {\vec{x},t} \right) - f_{\alpha }^{eq} \left( {\vec{x},t} \right)} \right)$$

20 $${\tilde{\text{g}}}_{{\upalpha }} \left( {\vec{x},t} \right) = {\text{g}}_{\alpha } \left( {\vec{x},t} \right) - \frac{1}{{\tau_{c} }}\left( {{\text{g}}_{\alpha } \left( {\vec{x},t} \right) - {\text{g}}_{\alpha }^{eq} \left( {\vec{x},t} \right)} \right)$$

In these equations, $\tilde{f}_{\alpha }$ and ${\tilde{\text{g}}}_{{\upalpha }}$ following the impact, depict the density and temperature distribution functions, respectively. It's worth noting that the natural convection, MF, and radiative heat terms are all included to the right-hand side of the equations in this study.

Step 1: Streaming: The distribution functions travel toward the surrounding nodes in the directions of their respective velocities in this phase, which begins immediately after the collision step.

21 $$\tilde{f}_{\alpha } \left( {\vec{x} + \vec{e}_{\alpha } \delta t,t + \delta t} \right) = \tilde{f}_{\alpha } \left( {\vec{x},t} \right)$$

22 $${\tilde{\text{g}}}_{{\upalpha }} \left( {\vec{x} + \vec{e}_{\alpha } \delta t,t + \delta t} \right) = {\tilde{\text{g}}}_{\alpha } \left( {\vec{x},t} \right)$$

The following are some non-dimensional parameters:

23 $$\begin{aligned} Pr & = \frac{{\vartheta_{f} }}{{\alpha_{f} }},\;\,Be = \frac{{S_{g,T} }}{{S_{g} }},\;\,{ }Ra = \frac{{g\beta_{f} l^{3} \left( {T_{h} - T_{c} } \right)}}{{\alpha_{f} \vartheta_{f} }},\;\,Ha = B_{0} l\sqrt {\frac{{\sigma_{f} }}{{\rho_{f} \vartheta_{f} }}} \\ Rad & = \frac{{4T_{C}^{3} }}{{k_{f} }}\frac{{\sigma_{e} }}{{\beta_{R} }}\;\,\zeta = \frac{{\mu_{nf} T_{0} }}{{k_{f} }}\left( {\frac{{\alpha_{f} }}{{L\left( {T_{h} - T_{C} } \right)}}} \right)^{2} \\ \end{aligned}$$

In which, Pr, Be, Ra and Ha respectively are Prandtl, Bejan, Rayleigh and Hartmann numbers. Rad and ζ represent radiation parameter and irreversibility coefficient, respectively.

The GOE is as the following equation:

24 $$S_{g} = \underbrace {{\underbrace {{\frac{{{\text{k}}_{nf} }}{{T_{0}^{2} }}\left( {\left( {\frac{{\partial {\text{T}}}}{{\partial {\text{x}}}}} \right)^{2} + \left( {\frac{{\partial {\text{T}}}}{{\partial {\text{y}}}}} \right)^{2} } \right)}}_{{\begin{array}{*{20}c} {thermal\;Entropy} \\ {S_{gen,T} } \\ \end{array} }} + \underbrace {{\frac{{{\upmu }_{nf} }}{{T_{0} }}\left\{ {2\left[ {\left( {\frac{{\partial {\text{u}}}}{{\partial {\text{x}}}}} \right)^{2} + \left( {\frac{{\partial {\text{v}}}}{{\partial {\text{y}}}}} \right)^{2} } \right] + \left( {\frac{{\partial {\text{u}}}}{{\partial {\text{y}}}} + \frac{{\partial {\text{v}}}}{{\partial {\text{x}}}}} \right)^{2} } \right\}}}_{{\begin{array}{*{20}c} {friction\;Entropy} \\ {S_{{gen,{\text{F}}}} } \\ \end{array} }} + \underbrace {{\frac{{\upsigma _{nf} {\text{B}}^{2} }}{{{\text{T}}_{0} }}v^{2} }}_{{\begin{array}{*{20}c} {Magnetic\;feild\;entropy} \\ {S_{gen,} B} \\ \end{array} }}}}_{{S_{Total} }}$$

In the above equations, *k* is the thermal conductivity, *μ* is the viscosity. Moreover, subscription of *nf* refers to the nanofluid.

### Nanofluid properties relationships

The electrical conductivity, density, volumetric expansion coefficient, specific heat, and heat transfer coefficient of the nanofluid are calculated using the following equations, which show the electrical conductivity, density, volumetric expansion coefficient, specific heat, and heat transfer coefficient, respectively.

25 $$\upsigma _{{{\text{nf}}}} = \left( {1 - \upvarphi } \right)\upsigma _{{\text{f}}} + \upvarphi\upsigma _{{{\text{sn}}}}$$

26 $$\uprho _{{{\text{nf}}}} = \left( {1 - \upvarphi } \right)\uprho _{{\text{f}}} + \upvarphi\uprho _{{{\text{sn}}}}$$

27 $$({\uprho \beta })_{{{\text{nf}}}} = \left( {1 - \upvarphi } \right)({\uprho \beta })_{{\text{f}}} + \upvarphi ({\uprho \beta })_{{{\text{sn}}}}$$

28 $$\left( {\uprho {\text{c}}_{{\text{p}}} } \right)_{{{\text{nf}}}} = \left( {1 - \upvarphi } \right)\left( {\uprho {\text{c}}_{{\text{p}}} } \right)_{{\text{f}}} + \upvarphi \left( {\uprho {\text{c}}_{{\text{p}}} } \right)_{{{\text{sn}}}}$$

29 $$\upalpha _{{{\text{nf}}}} = \frac{{{\text{k}}_{{{\text{nf}}}} }}{{\left( {\uprho {\text{c}}_{{\text{p}}} } \right)_{{{\text{nf}}}} }}$$

In the above equations, the indices *f* and *sn* depict the nanoparticles and the base fluid, respectively. The thermal conductivity and viscosity of the nanofluid were determined using ref.^58^. The Brownian motion of nanoparticles is also taken into account in this model. With the aid of Ref.^59^, the thermal conductivity of the nanofluid is expressed as follows:

30 $${\text{k}}_{{{\text{nf}}}} = {\text{k}}_{{{\text{Static}}}} + {\text{k}}_{{{\text{Brownian}}}} = \frac{{{\text{k}}_{{{\text{sn}}}} + 2{\text{k}}_{{\text{f}}} - 2\left( {{\text{k}}_{{\text{f}}} - {\text{k}}_{{{\text{sn}}}} } \right)}}{{{\text{k}}_{{{\text{sn}}}} + 2{\text{k}}_{{\text{f}}} + \left( {{\text{k}}_{{\text{f}}} - {\text{k}}_{{{\text{sn}}}} } \right)\upvarphi }}{\text{k}}_{{\text{f}}} + 5 \times 10^{4}\upbeta \upvarphi\uprho _{{\text{f}}} \left( {{\text{C}}_{{\text{p}}} } \right)_{{\text{f}}} \sqrt {\frac{{{\text{kT}}}}{{\uprho _{{{\text{sn}}}} {\text{d}}_{{{\text{sn}}}} }}} {\text{f}}\left( {{\text{T,}}\upvarphi } \right)$$

The viscosity is also given with the help of reference^60^ as follows

31 $$\upmu _{{{\text{nf}}}} =\upmu _{{{\text{Static}}}} +\upmu _{{{\text{Brownian}}}} = \frac{{\upmu _{{\text{f}}} }}{{\left( {1 - \upvarphi } \right)^{2.5} }} + 5 \times 10^{4}\upbeta \upvarphi\uprho _{{\text{f}}} \left( {{\text{C}}_{{\text{p}}} } \right)_{{\text{f}}} \frac{{\upmu _{{\text{f}}} }}{{{\text{k}}_{{\text{f}}} {\text{Pr}}}}\sqrt {\frac{{{\text{k}}_{{\text{b}}} {\text{T}}}}{{\uprho _{{{\text{sn}}}} {\text{d}}_{{{\text{sn}}}} }}} {\text{f}}\left( {{\text{T,}}\upvarphi } \right)$$

In the above equations, the terms β and f (T‚ φ) are written as follows for the Al_2_O_3_/water nanofluid^61^.

32 $${\text{f}}\left( {{\text{T,}}\upvarphi } \right) = \left( {2.8217 \times 10^{ - 2} \upvarphi + 3.917 \times 10^{ - 3} } \right)\left( {\frac{{\text{T}}}{{{\text{T}}_{0} }}} \right) + \left( { - 3.0669 \times 10^{ - 2} \upvarphi - 3.91123 \times 10^{ - 3} } \right)$$

33 $$\upbeta = 8.4407\left( {100\upvarphi } \right)^{ - 1.07304}$$

Table 1 summarizes the characteristics of nanoparticles and water.

**Table 1:** Thermophysical properties of Al_2_O_3_/water nanofluid^23^.

	*C*_*p*_ (J/kg K)	*k* (W/m K)	*ρ* (kg/m^3^)	µ (kg/m s)	*σ* (Ω m)^−1^	*d*_sn_ (nm)
Water	4179	0.613	997.1	0.001	0.05	–
Al_2_O_3_	765	40	3970	–	10^−12^	47

### Thermal and hydrodynamic boundary conditions

The boundary conditions are shown in non-dimensional form applying non-dimensional parameters in Table 2. It is worth noting that U = V = 0 holds on all the walls due to the no-slip condition.

**Table 2:** Non-dimensional boundary conditions.

Left wall	X = 0	$0 \le {\text{Y}} \le {\text{H}}$	$\frac{\partial \theta }{{\partial {\text{X}}}} = 0$
Right wall	X = H	$0 \le {\text{Y}} \le {\text{H}}$	$\frac{\partial \theta }{{\partial {\text{X}}}} = 0$
Up wall	$0 \le {\text{X}} \le {\text{H}}$	Y = H	$\theta = 0$
Bottom wall	$0 \le {\text{X}} \le {\text{H}}$	Y = 0	$\frac{\partial \theta }{{\partial {\text{Y}}}} = 0$
Hot pipe	$X^{2} + Y^{2} = R^{2}$		$\theta = 1$

**Figure 2:**
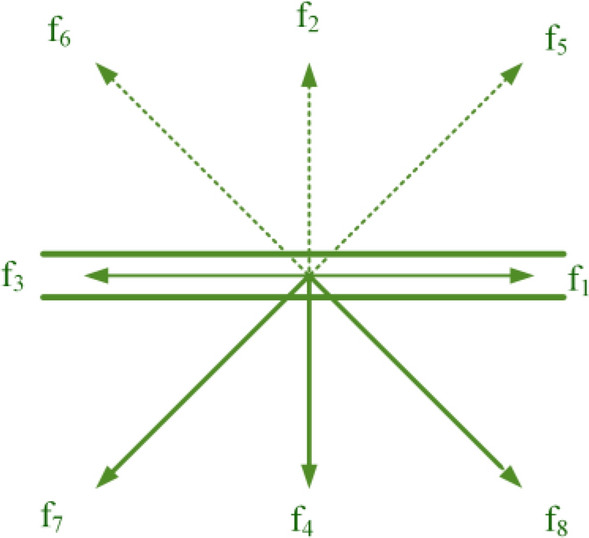
Boundary condition of mirror reflection.

Figure 2 shows how to apply the mirror reflection boundary conditions. The distribution functions specified by the dashed line are unknown functions.

Accordingly, the indeterminate functions of Fig. 2 are determined by defining the mirror reflection boundary conditions of Eq. (34), which can be generalized to other boundaries.

34 $$\begin{aligned} f_{2} & = f_{4} \\ f_{5} & = f_{7} \\ f_{6} & = f_{8} \\ \end{aligned}$$

The constant temperature boundary condition is also defined in Eqs. (35)^52^.

35 $$\begin{array}{*{20}l} {\text{Hot wall}} \hfill & {\quad {\text{Cold wall}}} \hfill \\ {g_{1} = \left( {T_{h} \left( {w_{1} + w_{3} } \right)} \right) - g_{3} } \hfill & {\quad g_{3} = \left( {T_{c} \left( {w_{1} + w_{3} } \right)} \right) - g_{1} } \hfill \\ {g_{5} = \left( {T_{h} \left( {w_{5} + w_{7} } \right)} \right) - g_{7} } \hfill & {\quad g_{7} = \left( {T_{c} \left( {w_{5} + w_{7} } \right)} \right) - g_{5} } \hfill \\ {g_{8} = \left( {T_{h} \left( {w_{8} + w_{6} } \right)} \right) - g_{6} } \hfill & {\quad g_{6} = \left( {T_{c} \left( {w_{8} + w_{6} } \right)} \right) - g_{8} } \hfill \\ \end{array}$$

The following equations represent the Nusselt number:

36 $$Nu = \frac{hL}{{k_{f} }}$$

37 $$h = \frac{{q_{\omega } }}{{T_{h} - T_{c} }}$$

38 $$q_{\omega } = k_{nf} \left( {\frac{\partial T}{{\partial X}}} \right)$$

39 $$Nu = - \frac{{k_{nf} }}{{k_{f} }}\left( {\frac{\partial \theta }{{\partial X}}} \right)$$

40 $$Nu_{Mid} = \frac{1}{L}\mathop \smallint \limits_{0}^{L} Nu dX = - \frac{1}{L}\frac{{k_{nf} }}{{k_{f} }}\mathop \smallint \limits_{0}^{L} \left( {\frac{\partial \theta }{{\partial X}}} \right)dX$$

## Mesh independence and validation

**Figure 3:**
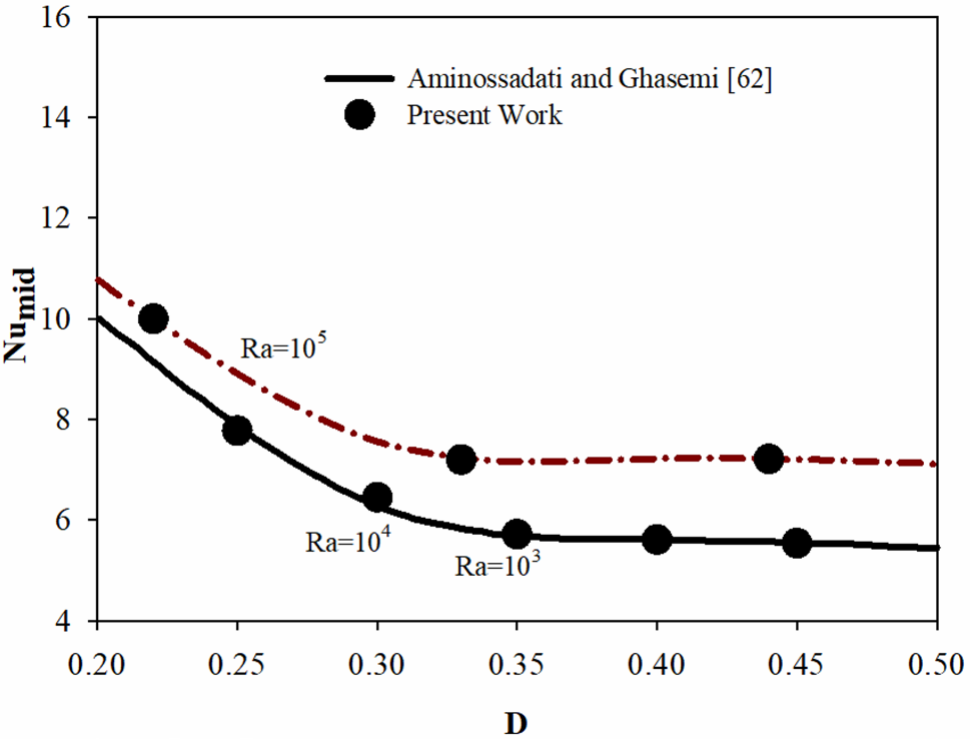
Average Nu obtained by present work compared that from Aminossadati and Ghasemi ^62^ .

The results in this paper have been validated using several papers, two of which are mentioned here. The average Nusselt number along the heated bottom wall at the cavity floor was compared to the results of Aminossadati and Ghasemi^62^ for NCHT inside a square cavity in the first comparison, displayed in Fig. 3. This cavity is being heated from below, and the vertical walls are at a cold temperature. As displayed in Fig. 2, the Nu values of the two works overlap with a good approximation.

The findings of this study are compared to those of Ghasemi et al.^63^ in the second comparison. In the problem studied by Ghasemi et al. the cavity is exposed to a MF. This comparison includes the effects of the MF as well as the nanoparticles. In Table 3, the amounts of the average Nu have been compared for a Ra of 10^5^ and a Hartmann number of 60. The results are seen to be in good agreement.

**Table 3:** Comparison of the average Nu between the present work and that of Ghasemi et al.^63^ at different nanoparticle volume fractions.

Φ	0.0	0.02	0.04	0.06
Ghasemi et al.^63^	1.850	1.830	1.814	1.807
Present work	1.838	1.851	1.871	1.808
%Err	0.6	1.1	3.1	0

Since the obtained values need to be independent of the grid points number, this section studies the influence of the points number on the average Nu and the generated entropy. The results for $Ha = 20$, $\phi = 0.03$, and $Ra = 10^{5}$ are shown in Table 4. According to these results, for grid sizes larger than 140 × 140, the number of points does not significantly affect the results. Hence, the grid size is taken to be 140 × 140 in the present work.

**Table 4:** The number of grid points for $Ha = 20$, $\phi = 0.03$, and $Ra = 10^{5}$.

Grid	80 × 80	100 × 100	120 × 120	140 × 140	160 × 160	180 × 180
Nu_*mid*_	9.992	10.152	10.245	10.307	10.307	10.308
Ψ*max*	3.752	3.986	4.036	4.046	4.046	4.046
S_*g*_	10.845	11.031	11.198	11.227	11.227	11.229

## Results and discussion

The base values considered are $\upvarphi = 0.3, \;\lambda = 0^{ \circ } , \;Ra = 10^{5} ,\; \gamma = 45^{ \circ } ,$, *Ha* = 20, *Rad* = 1. In each section, only the given variables are changed, and the rest of the values remain constant. Thus, only the variables are mentioned in the figure captions, and repeating the fixed values has been avoided.

### Changes in the nanoparticle volume fraction and radiation parameter

**Figure 4:**
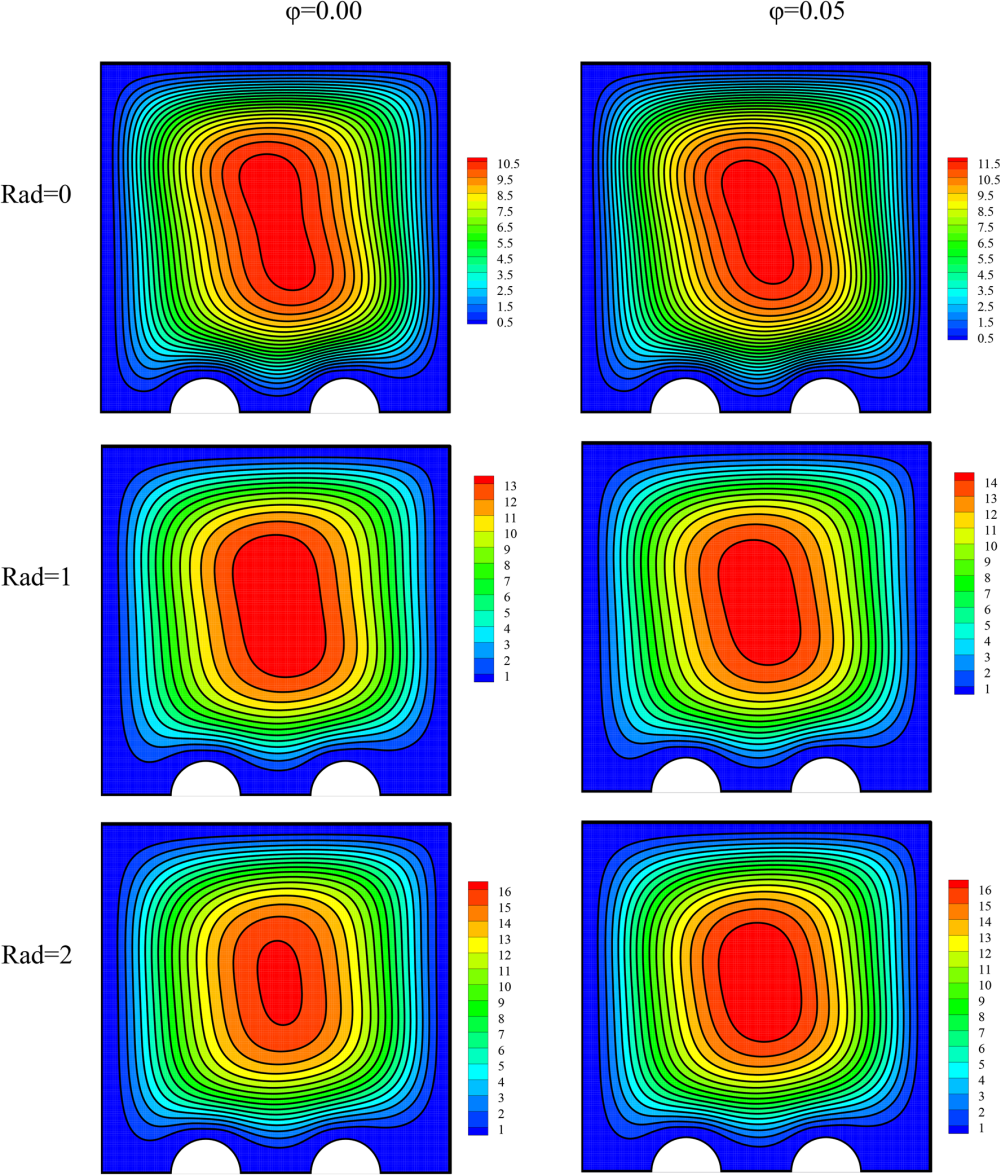
The flow field for a variable radiation parameter and different volume fractions at *λ* = 0°, *Ra* = 10^5^, *γ* = 45°, *Ha* = 20.

The flow field has been plotted in Fig. 4 for a variable radiation parameter, dissimilar volume fractions, and the mentioned constants. It is seen that adding nanoparticles to the base fluid augments the stream function levels. Being less than 10 percent, this intensification is not remarkable^64,65^. The rise in the thermal conductivity coefficient of the fluid with the addition of nanoparticles has augmented the flow speed. The addition of RHT to the cavity has also amplified the stream function levels. If present, RHT can lead to an overall improvement in heat exchange. An proliferation in either of the two studied parameters intensify the heat exchange. This improves NCHT between the fluid and the constant-temperature walls, ultimately leading to the temperature of the fluid adjacent to the constant-temperature walls approaching that of the walls. Hence, the temperature difference in the cavity rises, amplifying the buoyancy force. This ends in a rise in the fluid speed.

**Figure 5:**
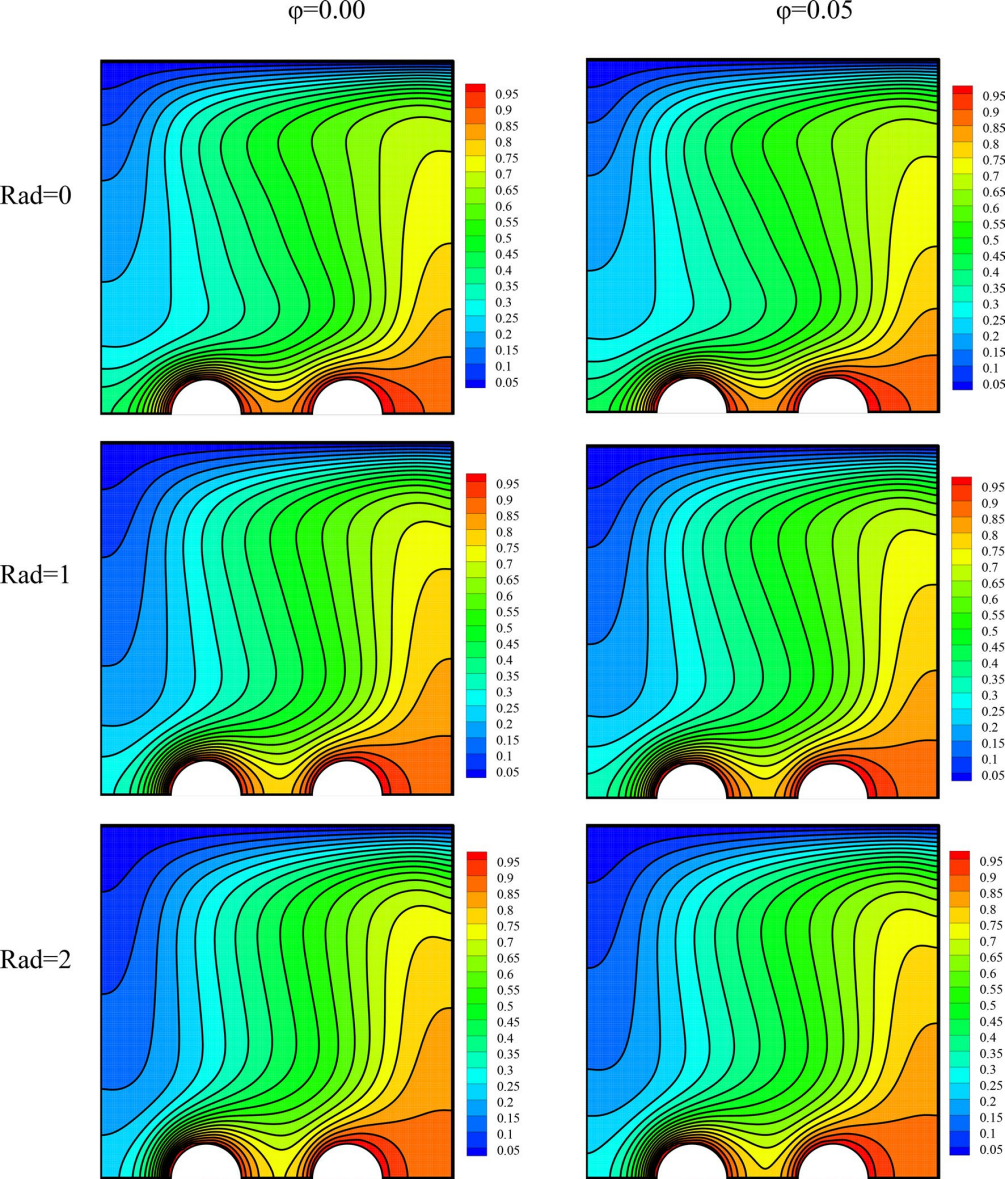
The temperature field for a variable radiation parameter and different volume fractions at *λ* = 0°, *Ra* = 10^5^, *γ* = 45°, *Ha* = 20.

The temperature field has been plotted in Fig. 5 for a variable radiation parameter and various volume fractions. Higher radiation and more nanoparticles in the fluid have both had small effects on the temperature fluid. The temperature field is strongly affected by the flow field.

**Figure 6:**
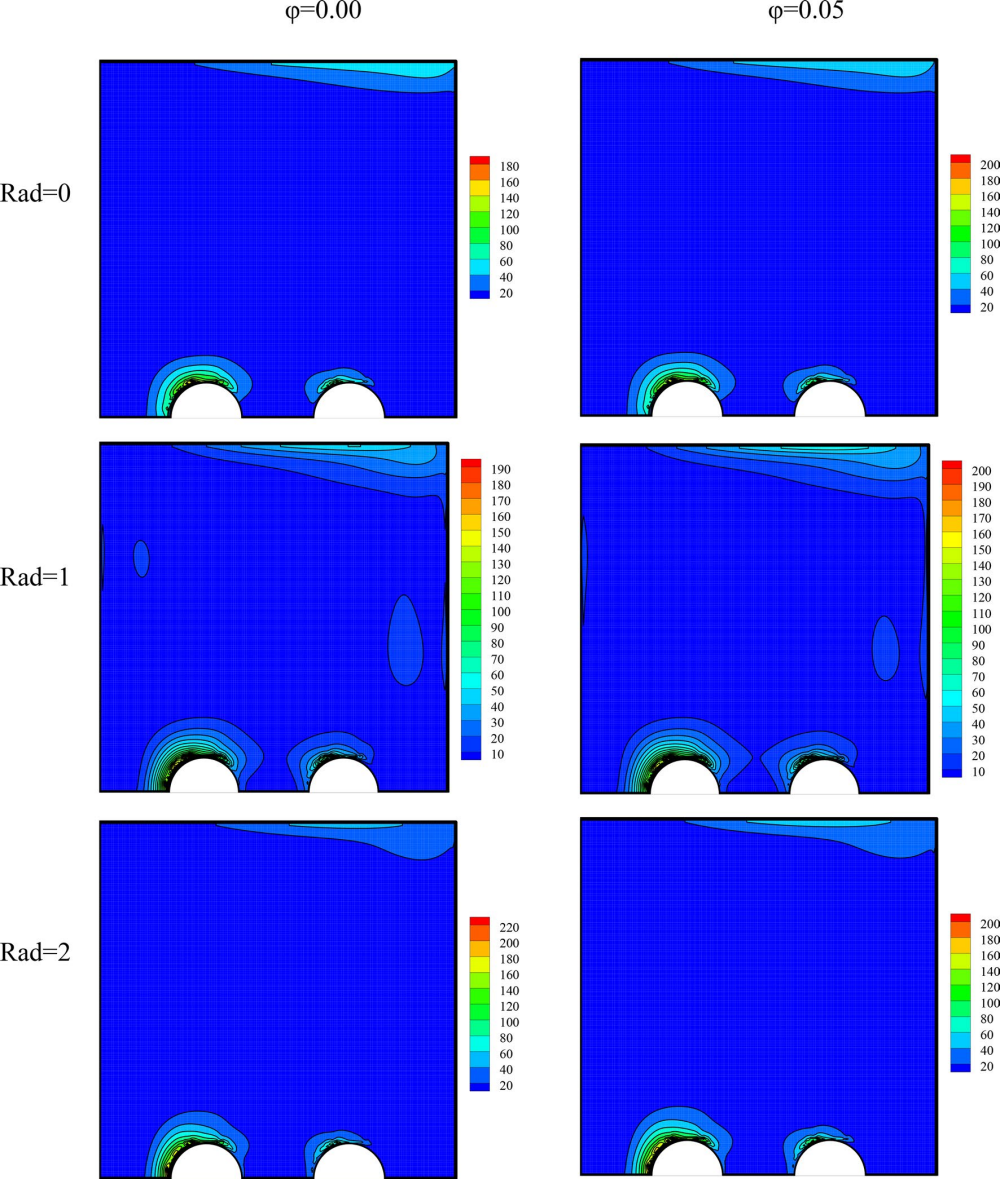
The entropy generation field for a variable radiation parameter and different volume fractions at *λ* = 0°, *Ra* = 10^5^, *γ* = 45°, *Ha* = 20.

The entropy generation field has been plotted in Fig. 6 for a variable radiation parameter and various volume fractions. The entropy generation field indicates the regions of high and low entropy generation. The entropy generation is low in most of the cavity. These regions are shown in blue and have low entropy generation. On the other hand, the regions shown in red are those with high entropy generation. In these regions, entropy generation is larger than in other regions possibly because of an enhancement in the temperature gradient or large changes in velocity. The regions with higher entropy generation are located where the temperature gradient is high, such as hot pipes where there are large temperature differences. Given the temperature gradient and the velocity, the level of entropy generation is different in this region. Intensifications in radiation and the number of nanoparticles in the fluid both cause a slight growth in the flow speed. Therefore, they somewhat rise the extent of changes in the velocity. Consequently, it is seen that with an escalation in these two parameters, high-entropy-generation regions gain higher entropy values.

**Figure 7:**
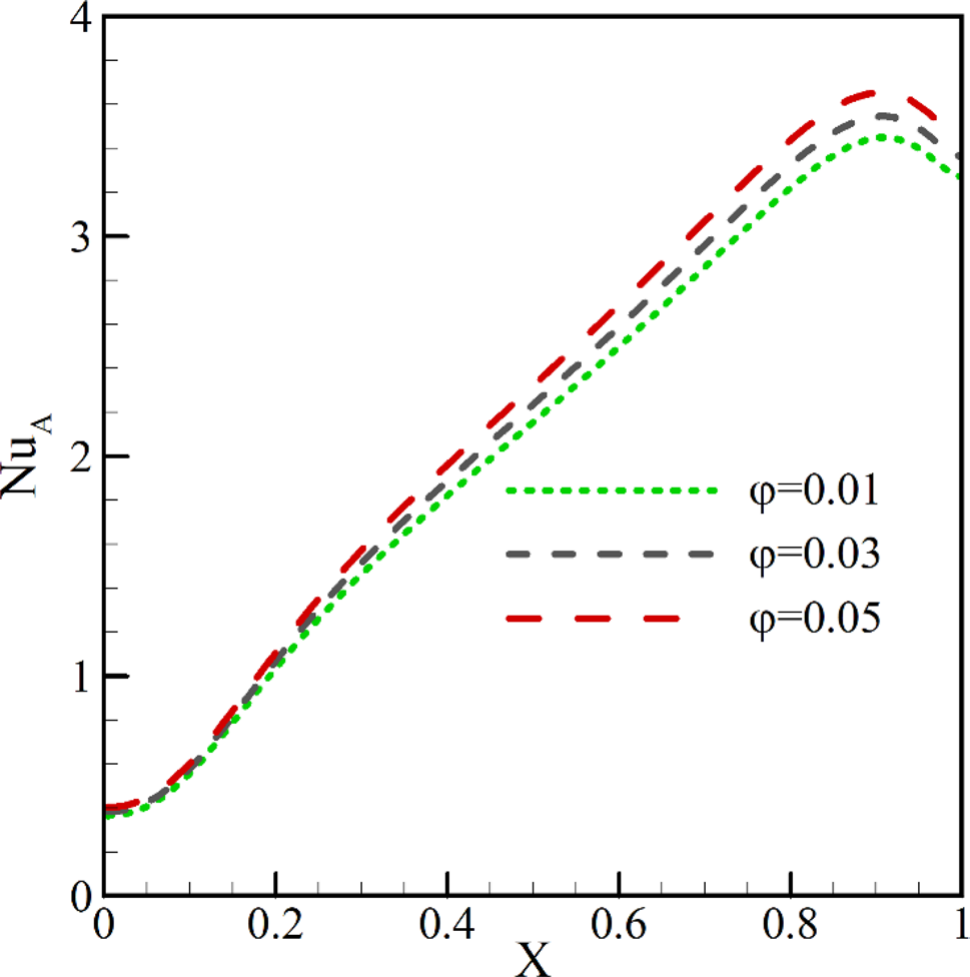
The average Nu for different volume fractions in the absence of radiation at *φ* = 0.3, *λ* = 0°, *Ra* = 10^5^, *γ* = 45°, *Ha* = 20.

The average Nu is plotted in Fig. 7 for dissimilar volume fractions in the absence of radiation. The addition of nanoparticles causes an enhancement in the fluid thermal conductivity. It also rises the viscosity of the fluid. Both of these parameters can significantly affect HTR.

A rise in viscosity leads to an intensification in shear stress and, hence, a decrease in the buoyancy force. Moreover, a higher thermal conductivity improves HTR from the walls to the fluid. In the end, it is seen in the graph that, for more concentrated nanoparticles, the Nu of the cold wall exhibits a dramatically by intensifying with a growth in the nanoparticle volume fraction. This growth is more in regions with a higher temperature gradient. Intensification in viscosity is especially significant in natural convection; thus, it has an imperative overall influence on HTR. For this reason, it is seen that the changes in the Nu with the addition of nanoparticles at small nanoparticle volume fractions are at first small and, then, rise.

The average Nu and total generated entropy for dissimilar radiation parameters are observed in Table 5. A proliferation in the radiation parameter is equal to a stronger RHT in the cavity. The rise in this mode of heat transfer causes the fluid to have larger overall heat exchange with the isothermal wall and hot pipes. Therefore, it is observed that with a rise in RHT, the average Nu on the cold wall and the overall average Nu in the cavity rise. Furthermore, the addition of this parameter has finally led to an augmentation in entropy generation. It was observed in the flow field that the flow speed rises with a rise in radiation in the cavity. Hence, speed changes in the cavity are more than before. This intensification causes the entropy generation to rise in the cavity, similar to the HTR.

**Table 5:** The average Nu and total generated entropy for dissimilar radiation parameters at *φ* = 0.3, *λ* = 0°, *Ra* = 10^5^, *γ* = 45°, *Ha* = 20.

Rad	0	1	2
Nu_Mid_	2.15	3.32	4.29
% Nu_Mid_	0	54.4	99.5
S_g_	7.33	8.25	9.08
% S_g_	0	12.5	23.8

### Variations in the Rayleigh number and the magnetic field angle

**Figure 8:**
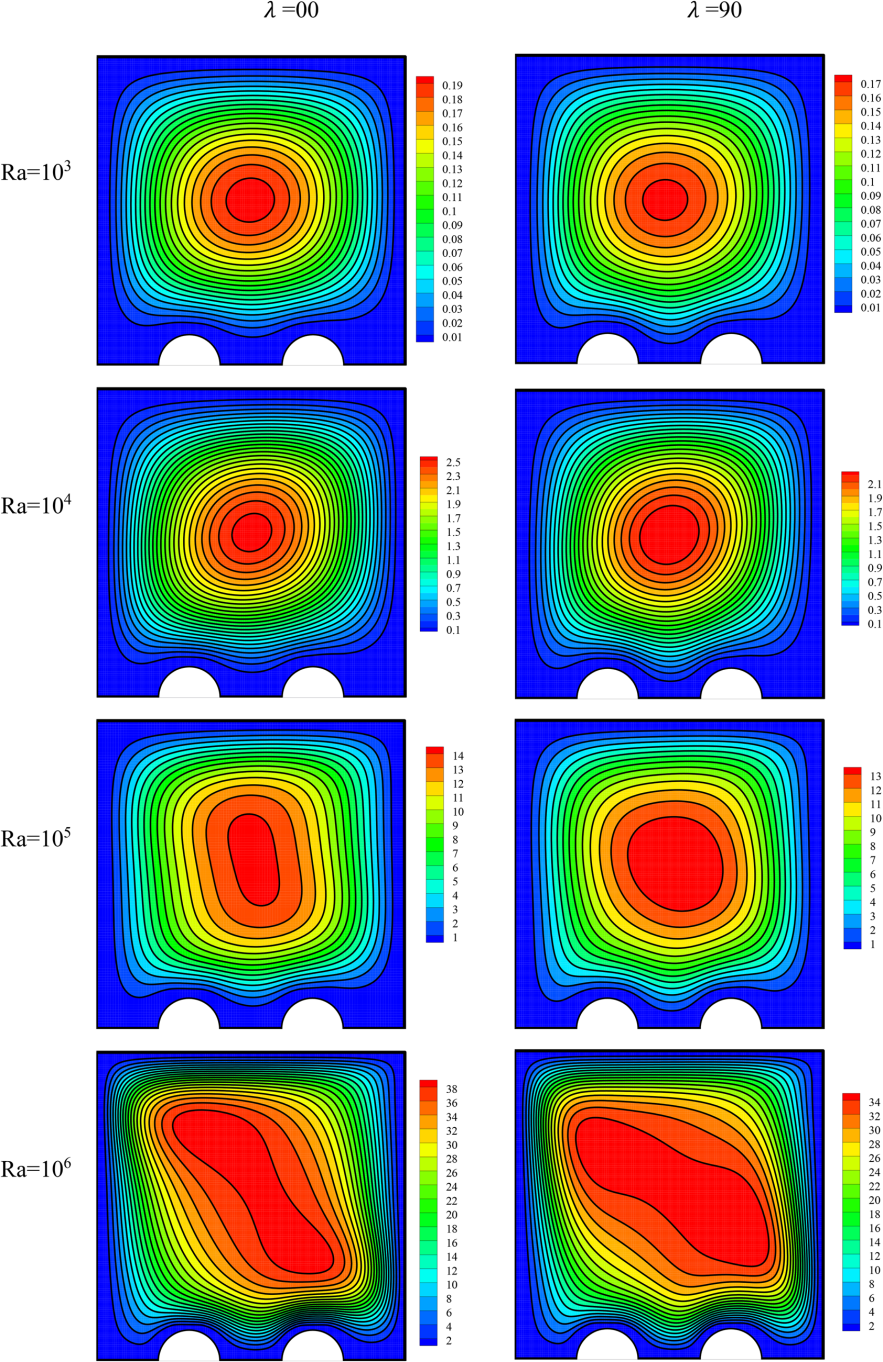
The flow field for varying values of Ra and MF angle at *φ* = 0.3, *γ* = 45°, *Ha* = 20, *Rad* = 1.

The flow field has been presented in Fig. 8 for varying values of Ra and MF angle. Similar to the majority of references, the intensification in the Ra has augmented the stream function level also in this research. A larger Ra causes intensification in the buoyancy force. Therefore, it intensifies the natural convection speed of the fluid. With the growth in this number, also the streamlines density intensifies close to the cavity walls and decreases in the middle of the cavity. This indicates large changes in speed in this region. In the region near the walls, changes in the speed are larger due to the no-slip condition and the formation of a boundary layer; these changes augment with an augmentation in the fluid speed. The MF angle has a direct effect on the direction of application of the Lorentz force on the cavity. Hence, it also affects the vortex shape, especially at larger Ra. It is observed that the maximum stream function levels corresponding to a MF angle of 0 are higher than those corresponding to a MF angle of 90°. The effect of the MF angle on the flow speed can be different depending on the problem conditions. In this case of special geometry, it is observed that a horizontal MF is more appropriate for the problem than a vertical one.

**Figure 9:**
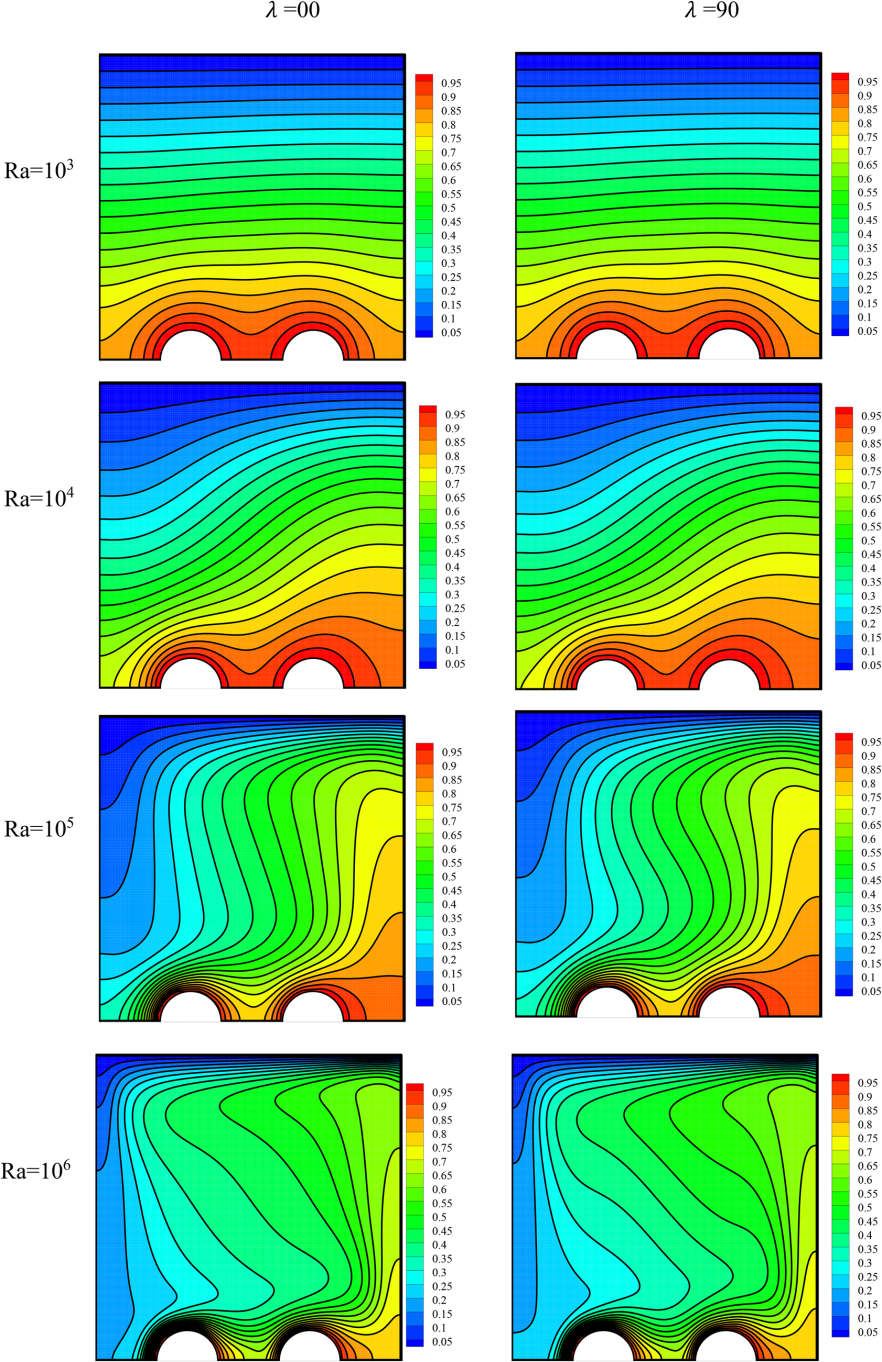
The temperature field for varying values of Ra and MF angle at *φ* = 0.3, *γ* = 45°, *Ha* = 20, *Rad* = 1.

The temperature field has been plotted in Fig. 9 for varying values of Ra and MF angle. The streamlines can show the heat transfer mode, including natural convection or conduction. In simple conduction heat transfer and when the fluid speed and displacement are very small, the isothermal lines are orderly and straight. This shows that there is a linear temperature profile between the walls that are at different temperatures. This case can be observed at low Ra in the temperature field. On the other hand, in NCHT, the isothermal lines are cluttered due to the continuous displacement of fluid, and the temperature profile is no longer linear for the same reason. This can also be clearly observed at high Ra, where the fluid flow speed is high. Moreover, this displacement causes the temperature gradient to rise in regions with temperature differences. Therefore, the temperature lines are observed to be more compact on the pipes and the cold wall at high Ra. Additionally, the effect of the MF is small due to the small level of changes in speed in the temperature field. Nevertheless, the line density in the temperature field is observed to be slightly higher for a MF angle of 0 than for one of 90°.

**Figure 10:**
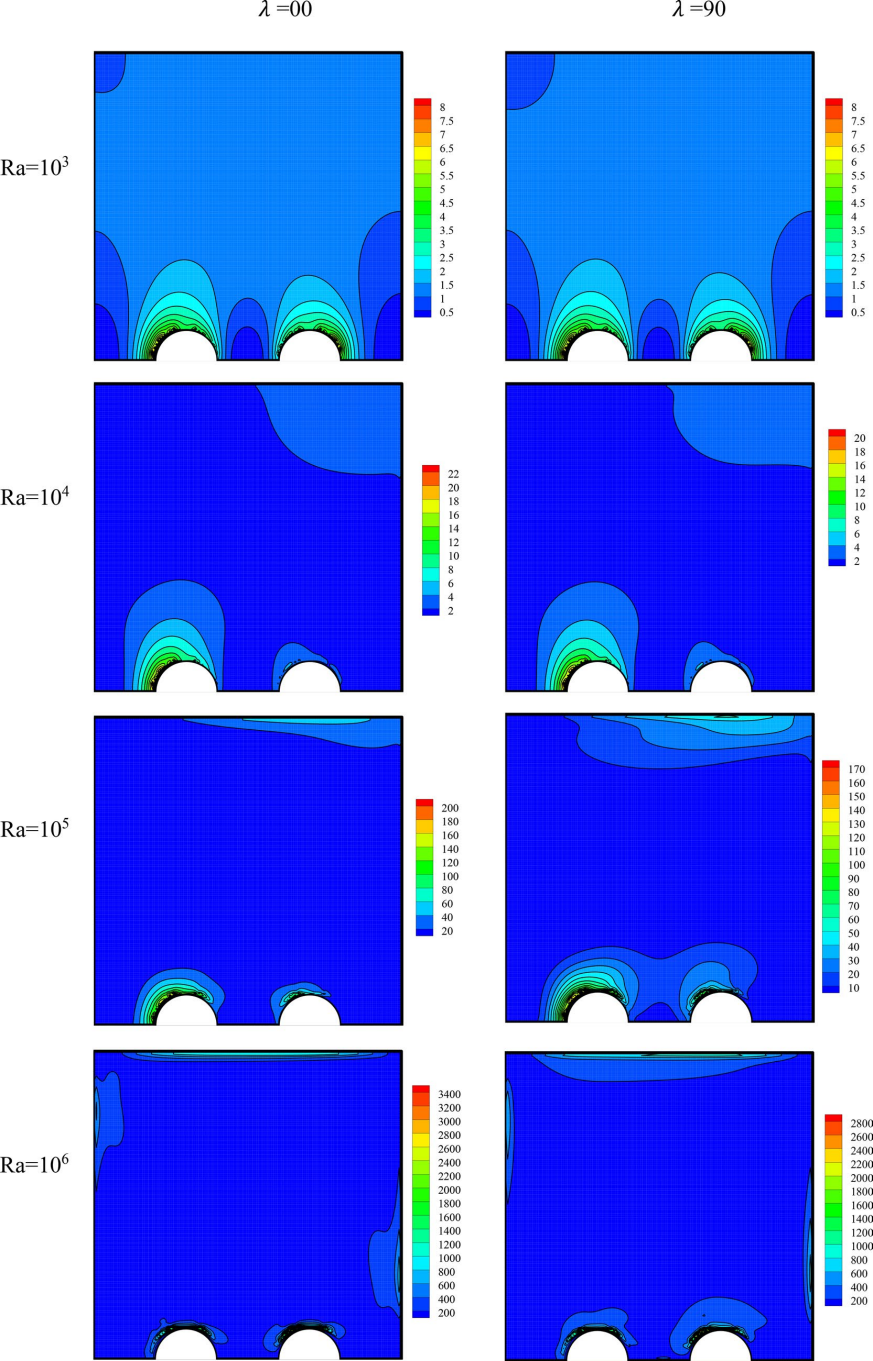
The entropy generation field for varying values of Ra and MF angle at *φ* = 0.3, *γ* = 45°, *Ha* = 20, *Rad* = 1.

The entropy generation field has been presented in Fig. 10 for varying amounts of Ra and MF angle. A rise in the Ra has augmented the speed and the density of the temperature lines. Thus, it will cause a change in the entropy level.

**Figure 11:**
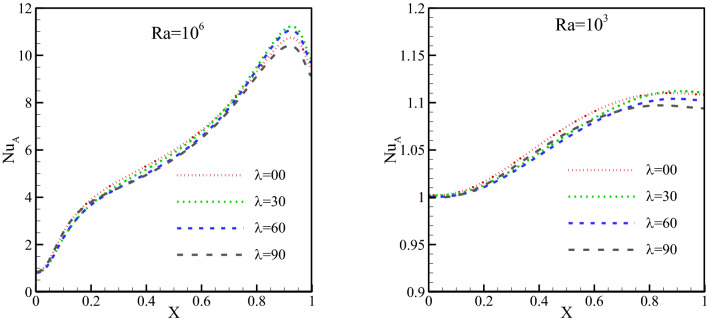
Local Nu on the cold wall for a Ra of (**a**) 10^3^ and (**b**) 10^6^ and different MF angles at *φ* = 0.3, *γ* = 45°, *Ha* = 20, *Rad* = 1.

The local Nu has been plotted at Fig. 11 for Ra of 10^3^ and 10^6^ and different MF angles. The local Nu has larger values at the beginning of the cold wall, where the gradient of temperature is greater, and decreases as one moves to the right side of the wall, where the temperature gradient reduces. This can be observed at any Ra and MF angle. The rise in the Ra has amplified the local heat transfer at every part of the cold wall. The reason has been a rise in the temperature difference due to a growth in vortex speed. The effect of the MF angle on the HTR is much smaller than that of the Ra. A rise in the MF angle reduces the HTR by only a small amount, especially at low Ra.

**Figure 12:**
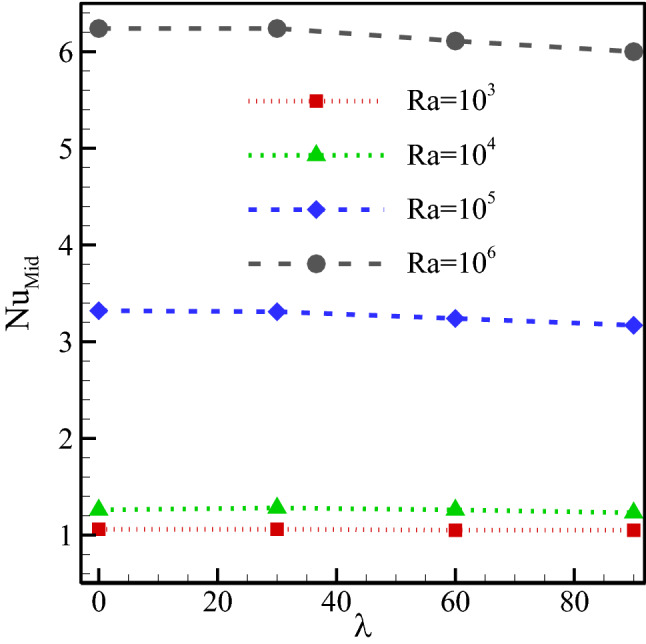
Average Nu for dissimilar Ra at *φ* = 0.3, *γ* = 45°, *Ha* = 20, *Rad* = 1.

The average Nu for dissimilar Ra has been plotted in Fig. 12. The intensification in Ra due to a rise in flow speed causes a higher temperature difference near the cold walls. Hence, HTR improves with the intensification of Ra, as clearly shown in the Nu graph. As seen in the flow field contours, the intensification of the MF angle caused a reduction in the fluid flow speed in the cavity. There, it also reduces the temperature gradient. Therefore, it is seen in the graph that the average Nu has decreased with a rise in the MF angle. Since the Ra has a larger impact than the MF angle on the flow speed, it is observed to have a larger effect on HTR here.

**Figure 13:**
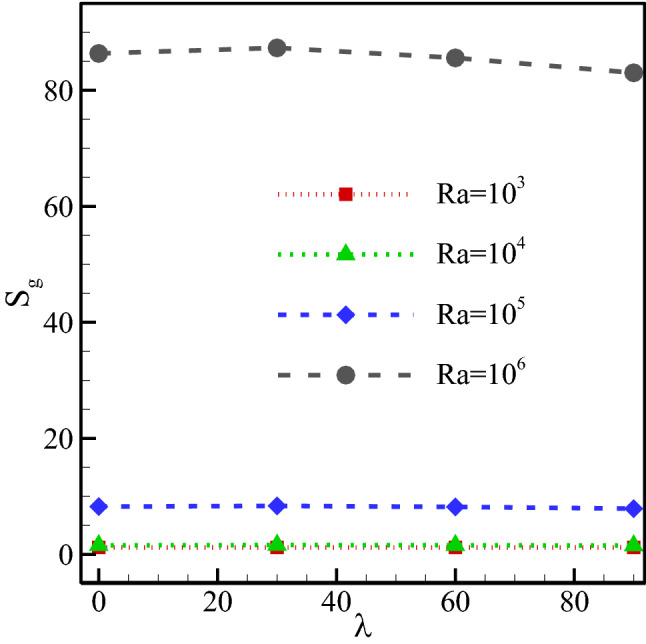
Total generated entropy for different Ra at *φ* = 0.3, *γ* = 45°, *Ha* = 20, *Rad* = 1.

The total generated entropy has been plotted in Fig. 13 for different Ra. Intensification in the speed and temperature difference, especially near the isothermal walls causes a rise in the generated entropy. This could be observed in the entropy contour. It was seen in the graph that the entropy generation strongly intensifies with an augmentation in the Ra. However, changes in entropy generation with the field angle are negligible compared to those with the Ra. The reason is the small changes in speed with the MF angle. Nevertheless, it is observed that, at a Ra of 10^6^, a proliferation in the MF angle slightly reduces the entropy generation.

## Conclusions

In this work, NCHT along with RHT of alumina/water nanofluid in a square chamber with an angle of 45° was studied. The chamber is affected by a MF with angle λ and the generated entropy is also considered. The following results have been obtained by changing the parameters of magnetic field angle, Ha, Rad and volume percentage of nanoparticles:

1. A rise in the radiation parameter from 0 to 1 increases the HTR by 45% and the generated entropy by 12.5%
2. Increasing the radiation parameter to 2 increases the Nusselt value by 99.5% and the entropy value by 23.8%.
3. Adding nanoparticles to the fluid increases HTR in the cavity.
4. An augmentation in the Ra leads to a growth in the average Nu and entropy generation, such that a rise in the Ra from 10^3^ to 10^6^ boosts HTR almost six-fold.
5. A rise in the MF angle reduces the HTR. This trend is especially prevalent at higher Ra.

## List of symbols

B_0_: Magnetic field strength
*C*: Lattice speed
$C_{p}$: Specific heat at constant pressure
*F*: External forces
*f*: Density distribution functions
$f_{eq}$: Equilibrium density distribution functions
*g*: Internal energy distribution functions
$g _{eq}$: Equilibrium internal energy distribution functions
*G*: Gravity
H: Dimensionless enclosure height
*Ha*: Hartmann number
*K*: The consistency coefficient
l: Enclosure height
l_1_: Radius of obstacle
L: Dimensionless thickness of obstacle
*Nu*: Nusselt number
*P*: Pressure
*Pr*: Prandtl number
R: Dimensionless radius of obstacle
*Ra*: Rayleigh number
Rd: Radiation parameter
S: Entropy
*T*: Temperature
*t*: Time
*u*: Velocity in x direction
*v*: Velocity in y direction
$$x,y$$: Cartesian coordinates

## Greek letters

$\sigma$: The electrical conductivity
$$\phi$$: Relaxation time
$$\tau$$: Shear stress
$\zeta$: Discrete particle speeds
$$\Delta x$$: Lattice spacing
$$\Delta t$$: Time increment
$$\alpha$$: Thermal diffusivity
$$\rho$$: Density
$$\mu$$: Dynamic viscosity
$\gamma$: Angle of cavity
$\lambda$: Angle of magnetic field
φ: Volume friction of nanoparticle

## Subscripts

$$C$$: Cold
f: Fluid
g: Generation
$$H$$: Hot
$$Mid$$: Average
$$x,y$$: Cartesian coordinates
$\alpha$: The number of the node
